# Does scrolling affect measurement equivalence of electronic patient-reported outcome measures (ePROM)? Results of a quantitative equivalence study

**DOI:** 10.1186/s41687-021-00296-z

**Published:** 2021-02-27

**Authors:** Saeid Shahraz, Tan P. Pham, Marc Gibson, Marie De La Cruz, Munther Baara, Sachin Karnik, Christopher Dell, Sheryl Pease, Suyash Nigam, Joseph C. Cappelleri, Craig Lipset, Patrick Zornow, Jeff Lee, Bill Byrom

**Affiliations:** 1 ICON PLC, South San Francisco, USA; 2 Pfizer, New York, USA; 3 Signant Health, Blue Bell, USA

**Keywords:** Patient-reported outcome, Patient-reported outcome measures, Intraclass correlation, Scrolling, BYOD, Measurement equivalence, Latin Square crossover design, ePRO, ePROM

## Abstract

**Background:**

Scrolling is a perceived barrier in the use of bring your own device (BYOD) to capture electronic patient reported outcomes (ePROs). This study explored the impact of scrolling on the measurement equivalence of electronic patient-reported outcome measures (ePROMs) in the presence and absence of scrolling.

**Methods:**

Adult participants with a chronic condition involving daily pain completed ePROMs on four devices with different scrolling properties: a large provisioned device not requiring scrolling; two provisioned devices requiring scrolling – one with a “smart-scrolling” feature that disabled the “next” button until all information was viewed, and a second without this feature; and BYOD with smart-scrolling. The ePROMs included were the SF-12, EQ-5D-5L, and three pain measures: a visual analogue scale, a numeric response scale and a Likert scale. Participants completed English or Spanish versions according to their first language. Associations between ePROM scores were assessed using intraclass correlation coefficients (ICCs), with lower bound of 95% confidence interval (CI) > 0.7 indicating comparability.

**Results:**

One hundred fifteen English- or Spanish-speaking participants (21-75y) completed all four administrations. High associations between scrolling and non-scrolling were observed (ICCs: 0.71–0.96). The equivalence threshold was met for all but one SF-12 domain score (bodily pain; lower 95% CI: 0.65) and two EQ-5D-5L item scores (pain/discomfort, usual activities; lower 95% CI: 0.64/0.67). Age, language, and device size produced insignificant differences in scores.

**Conclusions:**

The measurement properties of PROMs are preserved even in the presence of scrolling on a handheld device. Further studies that assess scrolling impact over long-term, repeated use are recommended.

## Introduction

Patient-Reported Outcome (PRO) measures have been increasingly gaining momentum in clinical outcome research because of recent movement toward patient-centeredness in both clinical practice and research [1, 2]. In the last two decades, the Food and Drug Administration (FDA) and European Medicines Agency (EMA) have progressively contributed to patient-focused drug development by requiring PRO endpoints in new drug applications [3] and including data from PROMs in drug labelling [4–6].

An increasing number of clinical research studies employ electronic formats to collect PRO measures (PROMs) in field-based and in-clinic settings [7]. This has been driven by the availability, low cost, and reliability of modern mobile devices such as smartphones and tablets, along with the requirement to improve the integrity and quality of data collected while limiting missing data entries and ensuring the timeliness of PROM completion [8]. Because most PROMs were originally developed and validated in pen-and-paper forms, migrating a PROM to an electronic format (ePROM) requires care to ensure the measurement properties of the original instrument are unaffected by the change in format [9].

Many clinical trials provide an electronic mobile device (provisioned device: PD) of a common make and model to all participants, to ensure that PROM presentation is identical for all participants. However, the drive to make clinical studies more patient-centric has led to increasing interest in collecting PROMs using the participants’ own device (bring your own device: BYOD) with the aim to make PROM collection more convenient. Due to smartphone screen size, ePRO solution providers typically aim to present a single PROM question per screen and to ensure all content is displayed without the requirement to scroll [10, 11]. When collecting PROMs using BYOD, the screen size and resolution of the participants’ devices may vary, and this may introduce the requirement for the user to scroll the screen to reveal both the question and response options for some or all PROM items.

Previous studies have provided some evidence on the equivalence of the PROMs after migrating from paper to electronic formats [12, 13]. However, past research examining the comprehension of information presented on computer monitors has reported mixed results when considering the impact of the requirement to scroll to retrieve information [14, 15]. One concern for studies utilizing ePROM is that a user may not review the complete question and response options before giving an answer to a questionnaire item with the presence of scrolling, and this behaviour may adversely affect the PROM measurement properties. While the measurement equivalence of PROMs comparing BYOD to PD has been studied [16], the impact of scrolling features on the response pattern associated with PROM completion has not been addressed. In this study, we aimed to evaluate the measurement equivalence of ePROMs in the presence and absence of scrolling on a set of provisioned smartphone devices as well as BYOD smartphones.

## Methods

### Design

**Fig. 1:**
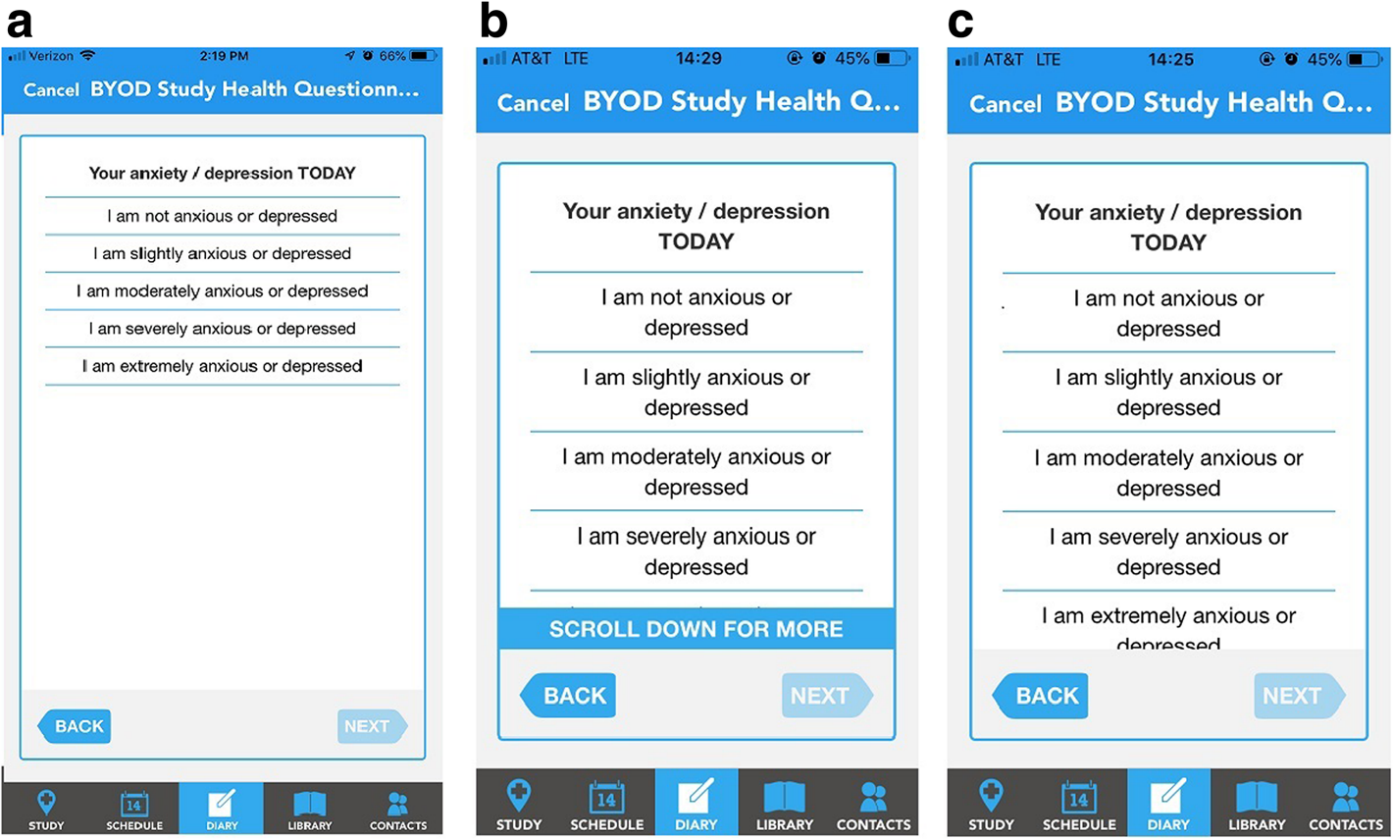
Format and layout differences for (**a**) no scrolling, (**b**) scrolling with the smart-scrolling feature, and (**c**) scrolling without the smart-scrolling feature

A Latin square crossover design enabling the randomization of four arms (sequences) and four periods (schedules) and balanced for first-order carryover was employed [17, 18]. This design incorporates blocks of 4 sequences of 4 individual administrations, with sequences randomly allocated within each block. Each sequence contains a single instance of each administration in such a way that within each block the treatment periods contain the same number of each administration, and individual administrations are preceded by each other administration the same number of times (balanced first order carryover). This particular design reduces errors as a result of imbalance contribution of the interventions and requires a relatively small sample size to conduct the trial. On each period, one of the following formats was administered: 1) A provisioned device not requiring scrolling (Samsung Galaxy J7: screen size: 5.5-in., screen resolution: 720 × 1280 pixels); 2) a provisioned device requiring scrolling to reveal all item text and including a “smart-scrolling” feature that disabled the “next” navigation button until all information was viewed (Samsung Galaxy Core Prime: screen size: 4.5-in., screen resolution: 480 × 800 pixels); 3) a provisioned device requiring scrolling (Samsung Galaxy Core Prime: screen size: 4.5-in., screen resolution: 480 × 800 pixels) without the smart-scrolling feature (user can advance without scrolling to reveal all information); and 4) BYOD (Android or iOS) with smart-scrolling. We provided no instruction to the participants regarding the type of Android or iOS mobile device that they could bring for use in the BYOD administration period The format layout differences and smart-scrolling feature are illustrated in Fig. 1. A washout period of 1 hour was used between each ePROM administration schedule. This washout period included a distraction task comprising a Paced Visual Serial Addition Test (PVSAT), developed using Apple Research Kit by ICON Clinical Research (Dublin, Ireland) and CRF Bracket (Arlington, VA). This task comprised a working memory addition test with numbers repeated every 3 s for 60 repeats, and was deployed on an iPad Mini device.

Included in the study was a mix of US English-speaking and US Spanish-speaking participants, aged 18 years and older, with a self-reported chronic medical condition causing daily pain or discomfort. Participants completed a selected set of PROMs. Study procedures were conducted at ICON’s office (Maryland, USA), with all participants being recruited from the US District of Columbia metropolitan area by Shugoll Research (Bethesda, USA) using their client database, referrals, and social media. All participants provided written informed consent. Salus Institutional Review Board (Austin, TX) provided ethical approval for the study. Participants were randomized to an administration schedule according to a pre-defined randomization list. Participants received training on use of the provisioned electronic smartphone devices from research staff to complete the PROMs.

The PROMs were delivered using the mProve Health ePRO platform (CRF Bracket, Arlington, VA). The ePRO platform was available in both US-English and US-Spanish versions, and participants were provided with the version corresponding with their primary language. The PROMs included the 12-Item Health Survey (SF-12) [19], EuroQol-5 Dimension- 5 Level (EQ-5D-5L), EuroQol Visual Analog Scale (EQ-VAS) [20–23], and three items measuring pain over the past week: a visual analogue scale (VAS), an 11-point numeric rating scale (NRS), and a 7-point Likert scale (LIK). The electronic implementation of the SF-12 and EQ-5D instruments were approved by the license holders, and the VAS, NRS, and LIK for pain were implemented according to ePRO design best practices [24]. Information was collected from participants on their attitudes towards BYOD use, along with familiarity with smartphone devices, by administering an end-of-study questionnaire on paper. The ePRO platform was configured such that no item could be skipped. However, it was possible that the participant could withdraw from the study during schedule or after finishing a schedule. These participants were excluded to ensure a balanced crossover design. Hence, missing information was only possible at schedule level and not at item level. However, we only included the participants who completed all four schedules.

To calculate the required sample size, we assumed 80% power with a one-sided alpha significance of 0.05 and a true underlying Intraclass Correlation (ICC) of 0.85. We further assumed the difference we wished to equate at least a lower bound for ICC of 0.70 [7, 9]. Subsequently, the required sample size per arm of the study was calculated to be 26 subjects. To compensate for losing five degrees of freedom as a result of extra variables in the model, we added 5 to the initial sample size (*N* = 31). The target recruitment sample size of 165 participants (assuming 25% dropout) was determined to provide 124 fully evaluable subjects with approximately 31 participants per sequence. We used the formula offered by Walter et al. to calculate the sample size [25]. No power analysis was performed for the logistic regression assumptions; however, we used a two-sided alpha at 0.05 as the significance level to interpret the results of the logistic regression analysis.

### Statistical analysis

Analyses were conducted using SAS 9.4 (SAS Institute, Inc., NC, USA), Stata 15 (StataCorp LLC, College Station, TX), and SPSS 25 (IBM, Armonk, NY). Mixed-effects generalized linear models (ME-GLM) were employed to fit the data and test the association between the treatment variables (e.g. scrolling vs. non-scrolling) with each PRO score. A random intercept model with study participants treated as random effects was specified with all the covariates (schedules and sequence of administration) modelled as fixed effects. ICCs were calculated using the method specified by McGraw & Wong to derive ICCs with 95% confidence interval. ICC (A, K) for a two-way mixed effects model with absolute agreement among more than two experiments (here schedules) was applied [26] to the PROMs. Additionally, the ICCs were calculated by dividing the variance of the random intercept by the total variance of the ME-GLM model, which is the sum of variance for the random intercept and that of the error term. The 95% confidence interval was obtained using the “delta method” [27, 28]. The more conservative method of estimating ICC (the one with a lower estimate) was eventually used as the primary method. Measurement equivalence was considered when a lower bound of the 95% Confidence Interval for the estimated ICC was at least 0.70 [7, 9]. The results on post-estimation ICCs were compared between two software applications, SAS 9.4 and STATA 15 for consistency.

Sensitivity analyses were conducted to examine differences between participants with any missing schedules and those who completed all four schedules. We fitted logistic regression models in which sex and age groups were set as the predictor variables and schedule completion status was set as the outcome variable. We also generated ICCs using all information (complete schedules and missing schedules) as well as only-complete schedules to evaluate the difference in the results given the input. Statistical significance was calculated for the two-sided 0.05 level throughout.

## Results

### Participants

**Fig. 2:**
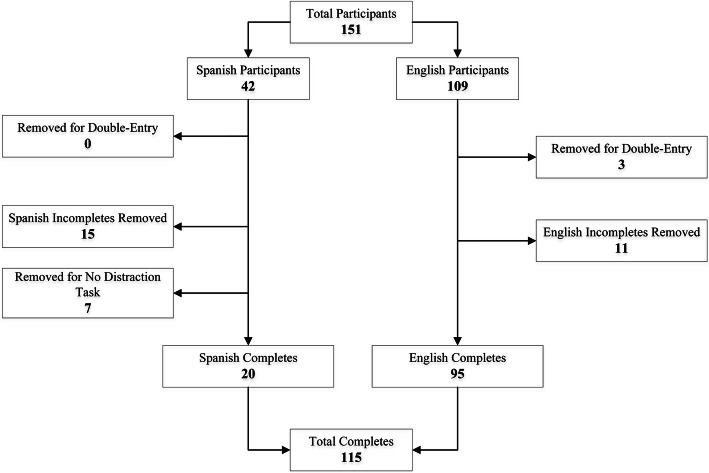
The flow of data gathering completion

Of the 151 eligible participants (42 US Spanish-speaking and 109 US English-speaking) initially recruited, 36 participants were excluded from the analysis for reasons described in Fig. 2. The final analyses included 115 participants (95 English-speaking and 20 Spanish-speaking), aged 21 to 75 years who completed all four schedules. Table 1 conveys detailed information on demographic features of the participants included in the final analyses. The most common self-reported cause of pain was arthritis (33.9%) followed by back pain (13%). Approximately 41% (*N* = 47) of the participants reported a heterogeneous array of reported morbidities, including diabetes. For all reported morbidities, only an indirect causal link between the reported morbidity and chronic pain was conceivable.

**Table 1:** Demographics and health conditions of participants

Variable ^**[a]**^	Total (***N*** = 115)
**Age (years)**	
Mean (SD)	52.1 (15.0)
Median	51
Min - Max	21–75
**Age Category**	
18–44 years	39 (33.9%)
45–64 years	37 (32.2%)
65+ years	39 (33.9%)
**Gender**	
Male	48 (41.7%)
Female	67 (58.3%)
**Race**	
Black	26 (22.6%)
Asian	5 (4.3%)
White	58 (50.4%)
Other	9 (7.8%)
Missing	17 (14.8%)
**Ethnicity**	
Hispanic or Latino	21 (18.3%)
Not Hispanic or Latino	77 (67.0%)
Missing	17 (14.8%)
**Language**	
Not bilingual Spanish speaker	95 (82.6%)
Bilingual Spanish speaker	20 (17.4%)
**Education**	
Did not complete high school/High school diploma/Technical training	5 (4.3%)
Some college	21 (18.3%)
2-year Associate’s degree/4-year Bachelor’s degree	46 (40.0%)
Master’s degree/Doctorate/Professional degree	43 (37.4%)
**Health Conditions**	
Arthritis	39 (33.9%)
Back Pain	15 (13.0%)
Headache	9 (7.8%)
Musculoskeletal pain	5 (4.3%)
Other	47 (40.9%)
**Have Difficulty Walking**	56 (48.7%)
**Have Problems Washing/Dressing**	20 (17.4%)
**Have Problems Doing Usual Activities**	68 (59.1%)
**Feeling Anxious/Depressed because of Health Condition**	78 (67.8%)

^a^Missing data included in calculation of percentages

### Familiarity with and attitudes toward BYOD

Table 2 provides further details about BYOD familiarity and preference and attitudes toward BYOD devices. Out of 115 participants, 66 (57.4%) participants used an Apple device and 49 (42.6%) used an Android device as the BYOD device in this study. Seventy-eight participants (67.8%) carried large devices, arbitrarily defined as one with a diagonal size at least 140 mm (5.5 in). Only two of the 115 participants reported inability to download and run the study app on the BYOD device and required assistance from this ePROM study’s research assistant to download the study app. Ninety-nine participants (86.1%) indicated that they were “definitely willing” to use a BYOD device for a clinical trial. Finally, 49 participants (57.4%) expressed that it was “essential/very important” that others could not see their data on their device.

**Table 2:** Patient familiarity, preferences, and attitudes towards BYOD devices

Variable^**a**^	Total (N = 115)
**BYOD mobile device type**	
Apple	66 (57.4%)
Android	49 (42.6%)
**BYOD mobile device size**	
Normal (< 140 mm or 5.5 in)	36 (31.3%)
Large (≥140 mm or 5.5 in)	78 (67.8%)
Missing	1 (0.9%)
**Able to download and run study app on own mobile device**	113 (98.3%)
**Familiar with downloading Apps**	115 (100%)
**Could you have downloaded this App on your own?**	
Definitely	90 (78.3%)
Probably	22 (19.1%)
Extremely Unlikely	3 (2.6%)
**Willing to use own device for a clinical trial?**	
Definitely	99 (86.1%)
Probably	10 (8.7%)
Possibly	4 (3.5%)
Extremely Unlikely	2 (1.7%)
**Had concern about using own device to download app to use in future**	8 (7.0%)
**Study reimburses for data charges**	
Essential	29 (25.2%)
Very Important	20 (17.4%)
Important	21 (18.3%)
A Little Important	10 (8.7%)
Not Important	35 (30.4%)
**Data cannot be seen by others on my device**	
Essential	47 (40.9%)
Very Important	19 (16.5%)
Important	14 (12.2%)
A Little Important	13 (11.3%)
Not Important	22 (19.1%)
**Easy to download and use App**	
Essential	46 (40.0%)
Very Important	32 (27.8%)
Important	21 (18.3%)
A Little Important	10 (8.7%)
Not Important	5 (4.3%)
Missing	1 (0.9%)
**App does not affect other apps on my device**	
Essential	80 (69.6%)
Very Important	21 (18.3%)
Important	5 (4.3%)
A Little Important	6 (5.2%)
Not Important	2 (1.7%)
Missing	1 (0.9%)
**App takes up a small amount of storage**	
Essential	37 (32.2%)
Very Important	28 (24.3%)
Important	26 (22.6%)
A Little Important	9 (7.8%)
Not Important	14 (12.2%)
Missing	1 (0.9%)
**Convenient to use own device instead of providing one?**	
More Convenient	39 (33.9%)
Neither More or Less Convenient	57 (49.6%)
Less Convenient	12 (10.4%)
Missing	7 (6.1%)
**Had Any concern about using own device to answer study questionnaire**	
Yes	7 (6.1%)
No	102 (88.7%)
Missing	6 (5.2%)

^a^Missing data included in calculation of percentages

### Measurement equivalence

Table 3 presents the mean (SD) for each scale or item score under each of the four schedules and provides estimated ICC (95% CI). Comparing the scrolling and non-scrolling schedules, the equivalence threshold criterion (a minimum of 0.7 lower band of the 95% confidence interval for ICC) was met for all scale/item scores except for the bodily pain scale score from SF-12, and usual activity and pain/discomfort items of the EQ-5D-5L. Estimated ICCs for SF-12 ranged between 0.72 and 0.96, and that for EQ-5D-5L items and scores ranged between 0.71–0.90. For the three pain scales, the ICCs showed a range between 0.81 and 0.95. The lower bound for 95% CI for bodily pain from SF-12 was 0.65 and for usual activity and pain/discomfort items of the EQ. 5D-5L was 0.67 and 0.64 respectively. The same pattern of success in meeting the measurement equivalence criteria was preserved for the overall ICC (a model with no comparison), contrasting BYOD schedule with non-scrolling schedule, and smart scrolling schedule versus non-smart scrolling schedule. The equivalence threshold criterion was met for eleven of twelve SF-12 items across all the three comparisons and for the overall estimated ICCs (results are not shown).

**Table 3:** Intra-class Correlations for questionnaire items between scrolling features

Scale/Subscale	Smart Scrolling	Without Smart Scrolling	Non-scrolling	BYOD	Overall	Scrolling vs. Non-Scrolling	BYOD vs Non-Scrolling	Smart Scrolling vs Without Smart Scrolling
	mean (SD)				Estimated coefficient of reliability, ICC (95% CI)			
**SF-12v2**								
General Health	46.35 (9.40)	46.65 (9.33)	46.14 (9.41)	46.68 (9.42)	0.96 (0.94, 0.97)	0.96 (0.94, 0.97)	0.94 (0.92, 0.96)	0.96 (0.94, 0.97)
Physical Functioning	42.96 (9.29)	43.03 (9.07)	42.21 (8.95)	42.96 (9.00)	0.92 (0.89, 0.94)	0.92 (0.89, 0.94)	0.91 (0.87, 0.94)	0.92 (0.89, 0.94)
Role Physical	44.03 (6.98)	43.15 (7.36)	42.82 (7.14)	43.59 (6.72)	0.81 (0.76, 0.86)	0.81 (0.76, 0.86)	0.80 (0.73, 0.86)	0.82 (0.76, 0.86)
Bodily Pain	44.71 (6.56)	44.63 (7.67)	45.34 (6.81)	45.89 (7.40)	0.72 (0.65, 0.78)	0.72 (0.65, 0.78)	0.77 (0.68, 0.83)	0.72 (0.65, 0.78)
Vitality	47.61 (8.76)	46.76 (8.67)	46.84 (8.75)	47.27 (8.70)	0.83 (0.78, 0.87)	0.83 (0.78, 0.87)	0.86 (0.80, 0.90)	0.83 (0.78, 0.87)
Social Functioning	45.30 (8.75)	45.53 (9.32)	45.15 (8.94)	45.38 (9.05)	0.84 (0.79, 0.87)	0.84 (0.79, 0.87)	0.83 (0.76, 0.88)	0.84 (0.79, 0.87)
Mental Health	46.85 (9.89)	46.70 (9.67)	46.60 (10.41)	46.65 (10.31)	0.90 (0.87, 0.92)	0.90 (0.87, 0.92)	0.91 (0.87, 0.93)	0.90 (0.87, 0.92)
Role Emotional	43.35 (10.43)	43.76 (9.88)	43.40 (10.45)	43.54 (10.17)	0.86 (0.81, 0.89)	0.86 (0.81, 0.89)	0.89 (0.84, 0.92)	0.86 (0.81, 0.89)
Mental Component Summary	46.69 (10.15)	46.75 (10.23)	46.63 (10.92)	46.54 (10.66)	0.93 (0.90, 0.94)	0.93 (0.90, 0.94)	0.94 (0.91, 0.96)	0.93 (0.90, 0.94)
Physical Component Summary	44.27 (7.52)	43.96 (8.04)	43.70 (7.53)	44.57 (7.86)	0.92 (0.89, 0.94)	0.92 (0.89, 0.94)	0.92 (0.89, 0.94)	0.92 (0.89, 0.94)
SF-6D Health Utility Index	0.67 (0.11)	0.67 (0.11)	0.67 (0.11)	0.68 (0.11)	0.90 (0.87, 0.93)	0.90 (0.87, 0.93)	0.93 (0.90, 0.95)	0.91 (0.88, 0.93)
**EQ-5D-5L**								
Index Value	0.76 (0.10)	0.76 (0.11)	0.76 (0.11)	0.77 (0.11)	0.86 (0.82, 0.90)	0.86 (0.82, 0.89)	0.89 (0.85, 0.93)	0.86 (0.82, 0.89)
EQ VAS	68.75 (17.43)	68.18 (17.54)	69.88 (17.06)	69.76 (18.46)	0.86 (0.81, 0.89)	0.86 (0.81, 0.89)	0.87 (0.82, 0.91)	0.85 (0.81, 0.89)
Mobility	1.73 (0.78)	1.76 (0.84)	1.71 (0.77)	1.74 (0.80)	0.85 (0.81, 0.89)	0.85 (0.81, 0.89)	0.89 (0.85, 0.92)	0.85 (0.81, 0.89)
Self-Care	1.46 (0.72)	1.37 (0.60)	1.43 (0.64)	1.40 (0.63)	0.77 (0.71, 0.82)	0.77 (0.71, 0.82)	0.85 (0.79, 0.89)	0.77 (0.71, 0.82)
Usual Activities	1.82 (0.66)	1.90 (0.72)	1.90 (0.71)	1.88 (0.69)	0.74 (0.67, 0.80)	0.74 (0.67, 0.80)	0.75 (0.66, 0.82)	0.74 (0.67, 0.80)
Pain / Discomfort	2.25 (0.66)	2.30 (0.69)	2.24 (0.68)	2.20 (0.64)	0.71 (0.64, 0.78)	0.71 (0.64, 0.78)	0.75 (0.66, 0.82)	0.72 (0.64, 0.78)
Anxiety / Depression	1.77 (0.77)	1.78 (0.81)	1.84 (0.89)	1.81 (0.84)	0.90 (0.87, 0.93)	0.90 (0.87, 0.93)	0.89 (0.85, 0.93)	0.90 (0.87, 0.92)
**0–100 VAS: Pain over the Past Week**	50.83 (21.32)	51.46 (22.64)	50.28 (21.10)	50.87 (22.34)	0.86 (0.82, 0.89)	0.86 (0.82, 0.89)	0.86 (0.80, 0.90)	0.86 (0.82, 0.89)
**11-Point NRS: Pain over the Past Week**	5.02 (2.13)	5.06 (2.12)	4.91 (2.16)	5.00 (2.15)	0.95 (0.93, 0.96)	0.95 (0.93, 0.96)	0.96 (0.95, 0.97)	0.95 (0.93, 0.96)
**7-Point Likert Scale: Pain over the Past Week**	3.83 (0.93)	3.94 (0.98)	3.82 (1.01)	3.83 (0.92)	0.81 (0.75, 0.85)	0.81 (0.75, 0.85)	0.79 (0.72, 0.85)	0.81 (0.75, 0.85)

Table 4 provides detailed information on the estimated ICC (95% CI) for the models for the covariate impact. The reliability threshold meeting success pattern remained unchanged for the impact of three covariates: language (Spanish versus English), device size (large versus normal), and age (45–64 years versus 18–44 years and 65^+^ versus 18–44 years). For all three covariate effects, the estimated ICCs ranged from 0.71 to 0.96 across all the PROMs. For bodily pain of SF-12, and usual activity and pain/discomfort of the EQ-5D-5L the lower band of the 95% CI for the ICCs ranged between 0.62 and 0.71 across all the PROMs.

**Table 4:** Intraclass Correlations for covariate impacts (language, device size, and age)

Scale/Subscale	Spanish Speakers vs. English Speakers		Normal BYOD vs. Large BYOD		45–64 vs. 18–44 Years old		65+ vs. 18–44 years old	
	Estimated coefficient of reliability, ICC (95% CI)	***p-***value	Estimated coefficient of reliability, ICC (95% CI)	***p-***value	Estimated coefficient of reliability, ICC (95% CI)	***p-***value	Estimated coefficient of reliability, ICC (95% CI)	***p-***value
**SF-12v2**								
General Health	0.96 (0.94, 0.97)	0.934	0.96 (0.94, 0.97)	0.313	0.96 (0.94, 0.97)	0.413	0.96 (0.94, 0.97)	0.580
Physical Functioning	0.92 (0.89, 0.94)	0.026	0.92 (0.89, 0.94)	0.368	0.91 (0.87, 0.93)	0.453	0.91 (0.88, 0.94)	0.085
Role Physical	0.81 (0.76, 0.86)	0.517	0.81 (0.76, 0.85)	0.109	0.81 (0.74, 0.86)	0.615	0.82 (0.76, 0.87)	0.782
Bodily Pain	0.71 (0.64, 0.78)	0.037	0.71 (0.64, 0.77)	0.029	0.72 (0.63, 0.79)	0.733	0.72 (0.63, 0.80)	0.651
Vitality	0.82 (0.76, 0.86)	0.003	0.83 (0.78, 0.87)	0.309	0.81 (0.74, 0.87)	0.163	0.82 (0.75, 0.87)	0.376
Social Functioning	0.84 (0.79, 0.88)	0.527	0.84 (0.79, 0.88)	0.992	0.82 (0.75, 0.87)	0.230	0.84 (0.78, 0.88)	0.162
Mental Health	0.90 (0.86, 0.92)	0.153	0.90 (0.87, 0.92)	0.525	0.89 (0.84, 0.92)	0.055	0.88 (0.83, 0.91)	0.014
Role Emotional	0.86 (0.81, 0.89)	0.622	0.86 (0.81, 0.89)	0.228	0.81 (0.74, 0.86)	0.028	0.84 (0.77, 0.88)	0.049
Mental Component Summary	0.93 (0.90, 0.95)	0.356	0.93 (0.90, 0.95)	0.595	0.91 (0.87, 0.94)	0.015	0.92 (0.88, 0.94)	0.005
Physical Component Summary	0.92 (0.89, 0.94)	0.147	0.92 (0.89, 0.94)	0.163	0.91 (0.87, 0.94)	0.372	0.91 (0.87, 0.93)	0.041
SF-6D Health Utility Index	0.90 (0.87, 0.93)	0.114	0.90 (0.87, 0.93)	0.312	0.89 (0.84, 0.92)	0.160	0.91 (0.87, 0.93)	0.137
**EQ-5D-5L**								
Index Value	0.86 (0.82, 0.89)	0.131	0.86 (0.82, 0.90)	0.317	0.87 (0.81, 0.90)	0.460	0.87 (0.82, 0.91)	0.891
EQ VAS	0.86 (0.81, 0.89)	0.553	0.85 (0.80, 0.89)	0.038	0.83 (0.77, 0.88)	0.587	0.88 (0.84, 0.92)	0.933
Mobility	0.85 (0.81, 0.89)	0.210	0.85 (0.81, 0.89)	0.378	0.87 (0.81, 0.91)	0.276	0.82 (0.75, 0.87)	0.026
Self-Care	0.77 (0.71, 0.82)	0.200	0.78 (0.72, 0.83)	0.712	0.78 (0.70, 0.84)	0.848	0.79 (0.72, 0.85)	0.617
Usual Activities	0.74 (0.67, 0.80)	0.321	0.74 (0.68, 0.80)	0.514	0.78 (0.71, 0.85)	0.386	0.71 (0.62, 0.78)	0.677
Pain / Discomfort	0.71 (0.64, 0.78)	0.254	0.72 (0.64, 0.78)	0.286	0.74 (0.65, 0.81)	0.341	0.71 (0.62, 0.79)	0.717
Anxiety / Depression	0.90 (0.87, 0.92)	0.202	0.90 (0.87, 0.93)	0.323	0.89 (0.85, 0.92)	0.277	0.88 (0.84, 0.92)	0.067
**0–100 VAS: Pain over the Past Week**	0.86 (0.81, 0.89)	0.046	0.86 (0.81, 0.89)	0.040	0.88 (0.83, 0.91)	0.710	0.82 (0.76, 0.87)	0.239
**11-Point NRS: Pain over the Past Week**	0.94 (0.93, 0.96)	0.048	0.95 (0.93, 0.96)	0.096	0.95 (0.92, 0.96)	0.517	0.91 (0.87, 0.94)	0.086
**7-Point Likert Scale: Pain over the Past Week**	0.81 (0.75, 0.85)	0.222	0.81 (0.75, 0.85)	0.674	0.79 (0.72, 0.85)	0.667	0.84 (0.78, 0.89)	0.852

### Sensitivity analysis

Cutting down the analytic sample from the full sample (*N* = 151) to the balanced sample (*N* = 115) trivially affected the ICCs and the confidence intervals. For instance, the overall ICCs for the SF-12-Physical Component Summary (PCS) score were estimated as 0.91(95% CI: 089–0.93) using the full sample and 0.92(95% CI: 0.89–0.94) after using the balanced sample. The equivalence analysis to obtain the ICCs with 95% CI was compared among SAS, STATA, and SPSS. SAS and STATA generated the exact results. However, by ignoring the covariate effect, SPSS consistently generated inflated ICCs. As an example, using SPSS the overall ICCs for SF-12 PCS score were calculated as 0.98 (0.97–0.98) using SPSS and 0.92 (0.89–0.94) using SAS and STATA. Mean differences of the scale/item scores across the four schedules using one-way analysis of variance and by including only the first administration (e.g., excluding administrations B, C, and D in ABCD sequence) were not statistically significant (one-sided *P*-value > 0.1 consistently).

## Discussion

The ePRO design good practice guidelines, such as those reported by the Critical Path Institute’s ePRO Consortium require the visibility of the full item stem text and its entire response options on the electric devices [24]. It follows that a principal concern in regard with migrating an existing pen and paper format PROM to an ePROM is that the participant may respond differently to items when the question and its response options are displayed fully compared to when items are partially displayed on a single screen. The difference in participants’ response patterns could theoretically stem from their unawareness of all the response options if some appear off screen. In addition, participants could find it inconvenient to scroll and, therefore, pick an item in view so that they can move to the next question quickly. For that reason, we examined the hypothesis that scrolling can alter participants’ response pattern.

This study provided a strong indication that the presence of scrolling is unlikely to affect PROM measurement properties. More specifically, we demonstrated measurement equivalence of the SF-12, EQ-5D-5L, and three different pain scales using common response scale types in the presence and absence of scrolling on provisioned and BYOD smartphone devices. There was measurement equivalence when comparing BYOD smartphones with non-scrolling provisioned devices satisfied the measurement equivalence. Similarly, measurement equivalence was preserved in comparing smart-scrolling with non-scrolling devices. Bodily pain scale score of the SF-12 and usual activity and pain/discomfort items of the EQ-5D-5L were the only scale/items which did not pass the measurement equivalence test. However, the lower band of the 95% confidence interval for the three pain scales exceeded the threshold of 0.7. Such inconsistencies may indicate discrepancies in item-level properties across different instruments that measure similar constructs. The impact of age, language, and smartphone size on the measurement equivalence was negligible and not statistically significant. The sensitivity analysis was done by preserving only the first administration, which converted the crossover design into a parallel design at the price of losing some power; however, the analysis of variance model showed no difference in mean scores of the PROMs across the four schedules. These sets of analyses supported the insignificant impact of the sequential testing on the measurement equivalence results.

It is noteworthy to emphasize that the focus of the current study was not to test the psychometric properties of these instruments on electronic devices. This study is meant to evaluate whether the changes in the question-answer display format on smartphone screens may result in changing the subject responses. A number of approaches are currently offered by ePRO solution vendors to mitigate the need for scrolling. One is to detect device features (make, model, etc.) on app installation and block devices that do not meet minimum size/specification criteria. Such an approach typically employs a look-up table of device specifications. While commercial databases exist, these have limitations, as it is hard to keep up to date with all makes and models (esp. Android) to enable this option for inclusion of all possible devices. A second method is to detect scrolling on a per-page basis and provide a scrolling indicator or disable navigation until scrolling has been accomplished (smart-scrolling). Finally, one can ensure that the navigation buttons are always at the foot of the page so the need to scroll to advance is required to reveal the entire questionnaire item before it is possible to advance to the next question. We utilized the smart-scrolling approach in this investigation.

In terms of design and analysis of the study, we employed a Latin square crossover design, which allowed the randomization of the four schedules and four different sequences. We followed previous research [7, 9] to select the acceptable lower band 95% confidence interval limit (i.e., 0.70 to serve as the equivalence threshold). The fixed one-hour distraction task between each subsequent pair of ePROM administration was assumed to effectively mitigate the participant’s recall of the response pattern from the previous administration to the next. By including two covariates, sequence and schedule, in the regression model for the equivalence estimation we tried to further mitigate the carryover effect.

The study comes with some limitations. While we were able to demonstrate measurement equivalence in the presence or absence of scrolling during repeated administration on a single day, we did not study the possible effects of scrolling during repeated use that is common with a typical clinical trial scenario. It would be valuable to study whether scrolling has a negative effect on completion compliance during longitudinal use, and whether response behaviour might be affected longitudinally if scrolling produces additional completion burden for the patient. Secondly, we only examined one method to mitigate scrolling, although it is likely that the other scrolling mitigation approaches would yield similar results. Finally, we had a small sample of participants who presented small BYOD smartphones and were not able to breakdown the sample for detailed analysis of the BYOD size effect. According to the latest data on smartphone sale by screen size, it is evident that small smartphones are still used by some people [29]. Hence, the results of this study on the impact of the BYOD size should be interpreted with caution.

## Conclusions

This study, to our knowledge, is the first research that evaluates scrolling providing some positive signals to help mitigate concerns over use of a scrolling feature when it is necessary. While the need for scrolling is unlikely on larger devices and can be completely prevented when providing a provisioned smartphone to study participants, the need to scroll cannot be completely eliminated in a BYOD setting where a pre-defined criteria to exclude small BYOD devices is not set up. Based on the results of our study, we make the following recommendations relevant to ePRO design in the future: 1) continue to design ePROMs to avoid scrolling when using a provisioned device; 2) mitigate scrolling by using one of the approaches described (smart-scrolling, scrolling indicator/pop-up, or navigation buttons at the foot of the screen requiring scrolling to progress), 3) over-ride certain user-adjusted screen display settings within the app display where possible; and 4) always provide partial provisioning as an option to allow for patients with unsuitable smartphones, which can be facilitated by defining a minimum specifications that can be easily identified by patient/site [9].

## Data Availability

The datasets used and/or analysed during the current study are available from the corresponding author on reasonable request.

## Abbreviations

*BYOD*: Bring your own device
*CI*: Confidence interval
*EMA*: European Medicines Agency
*ePRO*: Electronic patient reported outcome
*ePROM*: Electronic patient reported outcome measure
*EQ-VAS*: EuroQol Visual Analog Scale
*EQ-5D-5L*: EuroQol-5 Dimension- 5 Level
*FDA*: Food and Drug Administration
*ICC*: Intraclass correlation coefficient
*LIK*: Likert scale
*ME-GLM*: Mixed-effects generalized linear models
*NRS*: Numeric rating scale
*PCS*: Physical Component Summary
*PRO*: Patient-reported outcome
*PROM*: Patient reported outcome measure
*PVSAT*: Paced Visual Serial Addition Test
*SD*: Standard Deviation
*SF-12*: 12-Item Health Survey
*VAS*: Visual analogue scale
